# A Rapid Review of International Evaluations of interRAI Systems

**DOI:** 10.1177/08404704251389191

**Published:** 2025-11-10

**Authors:** K. Mathias, L. Martin, K. K. Alharbi, T. F. Smith, J. de Almeida Mello, K. Hermans, N. Vereker, M. de Stampa, O. Intrator

**Affiliations:** 1 153482 University of Waterloo, Waterloo, Ontario, Canada; 2 157330 Lakehead University, Thunder Bay, Ontario, Canada; 3 48058 Umm Al-Qura University, Makkah, Saudi Arabia; 4 226783 Nipissing University, North Bay, Ontario, Canada; 5 26657 KU Leuven, Leuven, Belgium; 6 8004 HSE, Cork, Ireland; 7 644617 Departmental Gerontological Center, Marseille, France; 8 Université de Versailles Saint-Quentin-en-Yvelines, Paris, France; 9 607692 Icahn School of Medicine at Mount Sinai, New York, New York, United States of America; 10 James J. Peters VA Medical Center, Bronx, New York, United States of America

## Abstract

The implementation of interRAI systems is often influenced by factors including healthcare priorities, policy and service requirements, fragmentation of care systems, and existing data standards. Utilizing a rapid review methodology, PubMed was searched for publications, and senior fellows in countries that implemented or piloted interRAI systems shared reports with a total of 40 papers retained and reviewed. Strengths, barriers, and recommendations were extracted. Comprehensive standardized instruments, solid psychometric properties, and the multiple uses of assessment information were noted as strengths. The most frequently cited barrier was lack of infrastructure, related to technological infrastructure and education/training. This review offers lessons to facilitate successful implementation of interRAI systems. Resource allocation to support hiring of staff, education/training, and the purchasing of information and technology solutions; and technology infrastructure to reduce the burden of assessment and support the continuity of care across care settings were among the top recommendations provided in this review.

## Introduction

**Figure 1.**
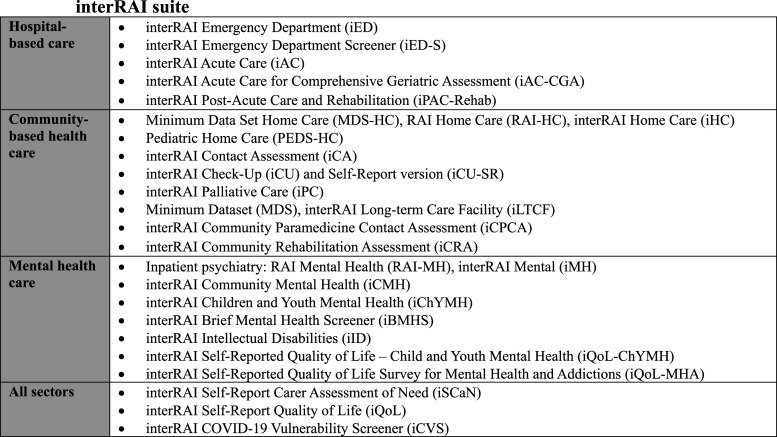
interRAI Suite

interRAI is a not-for-profit collaborative of researchers and practitioners from multiple countries dedicated to providing high quality information to care professionals, administrators, and policy-makers to assist in providing care to meet the needs of people receiving health and social services.^1^ To this end, a suite of integrated, standardized assessment instruments have been developed across the full spectrum of health services (e.g., community-based and hospital care) and age continuum (i.e., from infants and toddlers to older adults) (Figure 1). Standardized assessments are an essential part of health and social services because they help characterize the needs of patients and caregivers, support care planning, improve quality of care, track and evaluate health outcomes, and optimize resource allocation.^2^ As interRAI’s standardized assessments produce a variety of outputs (e.g., scales, planning protocols, quality indicators, and resource utilization groupings) to inform individual, organizational, and system-level decisions, many have been mandated nationally for use in several countries (e.g., Belgium, Canada, Finland, Hong Kong, Ireland, and New Zealand) and in many states in the United States (US).^1^

The implementation of interRAI systems has varied significantly across organizations, jurisdictions, and countries, often influenced by cultural climate, healthcare priorities, procedural mandates, government support, policy and service requirements, fragmentation of care systems between multiple funding agencies, and existing data standards.^3-5^

To understand the lessons learned and perspectives from international experiences, we conducted a rapid review to determine facilitators and barriers to implementation of interRAI systems. Based on this we developed a set of recommendations to support successful implementations of interRAI systems will be developed, including mitigation strategies for known barriers. These recommendations can support decision makers embarking on implementations of interRAI systems, for example, via their use as a checklist, in their consideration of funding and investment priorities, as well as in development of a communication strategy with staff.

## Methods

We followed the Preferred Reporting Items for Systematic Reviews and Meta-Analyses (PRISMA) criteria for this rapid review. A protocol guided this rapid review but was not registered, and ethics review was not required for conducting this review.

We searched the academic database PubMed through the University of Waterloo online library access. Search terms used in PubMed included (interRAI) AND (barriers OR facilitators OR challenges) AND (implement* OR adopt* OR use). Inclusion criteria for the PubMed search included persons across care settings, interRAI instruments or other instruments assessing health, function, social support, behaviour, quality of life and service use measures, studies that discussed strengths and barriers to implementing interRAI assessment systems, peer-reviewed articles, and only English-speaking studies. There were no restrictions on types of study designs with a mixture of qualitative and quantitative studies, the year of publication, or geographical location. Studies that did not discuss implementation of interRAI systems, non-English studies and letters, commentaries, and editorials which do not include relevant primary empirical data were excluded. The title, abstract, and full texts were screened by two independent reviewers for inclusion. When there was a conflict in the screening and reviewing of articles in Covidence, two other reviewers were consulted.

interRAI fellows were asked to provide information on any implementation-related reports they were aware of in their countries. The inclusion and exclusion criteria for reports did not differ than those for journal articles; all eligible reports were part of the data extraction process. Reports were screened by two independent reviewers and when relevance was questionable, the two reviewers discussed the paper to arrive at a conclusion. Reports in French and Dutch were translated into English and incorporated in the data extraction table.

We extracted the following into an excel spreadsheet: author, year, title, country; interRAI assessment and version; other instruments/systems reviewed; care setting; key findings; implemented (y/n); strengths for implementation; barriers to implementation; and recommendations/considerations for implementation. A thematic analysis of the papers was conducted to establish themes among the strengths and barriers to implementation. KM developed the initial themes based on the extracted information and LM reviewed them. Any issues were discussed and resolved by these authors.

## Results

**Figure 2.**
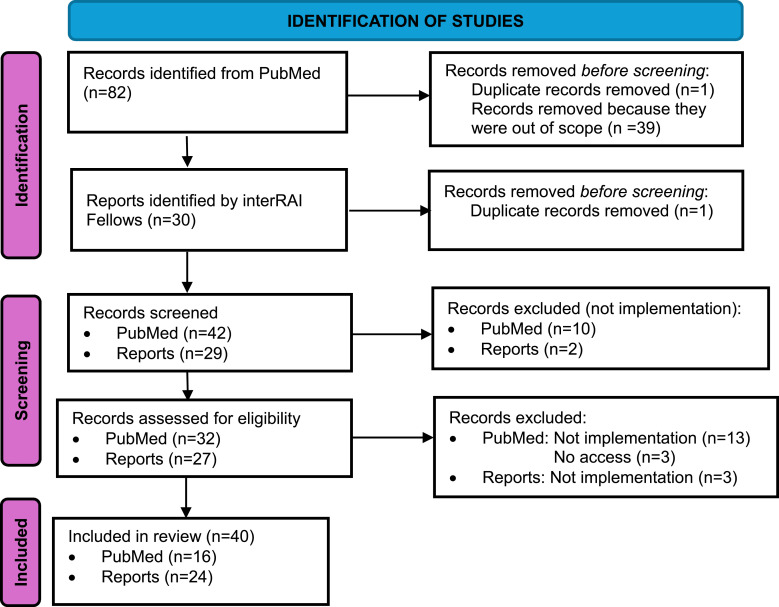
PRISMA Flowchart

Forty two articles and 29 reports were screened and after removal of duplicates, 32 articles and 27 reports were read in detail in full text, and 16 articles and 24 reports were included in the data extraction (Figure 2). Moving forward, “papers” is used to refer to articles and reports.

### Characteristics of Sources of Evidence

The majority of implementations studies were from Canada^6-19^ (n = 15), followed by Belgium^20-28^ (n = 9), Australia^5,29,30^ (n = 3), New Zealand^31-33^ (n = 3), and the United States^34-36^ (n = 3); some also originated from France,^37^ Hong Kong,^38^ Ireland,^39^ the Netherlands,^40^ and Switzerland.^41^ Two papers reported on implementations in multiple countries.^4,42^

#### interRAI Instruments and Care Settings

The home care instrument was the most studied overall (n = 21). It was reported on in the context of general home care, community-based care, home delivered meals program, and long-stay home care settings using early versions (Minimum Data Set-Home Care (MDS-HC)^10,22,32,38^ Resident Assessment Instrument-Home Care (RAI-HC)^6,11,17,34^) and the most recent versions (interRAI Home Care (iHC)^4,12,13,20,21,26,27,29,31,33,35,37,43^). Other instruments designed for use in home and community health settings included the Pediatric-Home Care (PEDS-HC),^15^ interRAI Check-Up (iCU),^4^ iCU-Self-Report (iCU-SR),^16,18^ interRAI Contact Assessment (iCA),^4,12,13,32^ and the interRAI Palliative Care (iPC).^4,13,23^

Eleven papers focused on the assessment of long-term care in residential aged care homes, nursing homes/long-term care homes, chronic care, rehabilitation hospitals, and complex continuing care hospital settings. Both the earlier versions (i.e., MDS 2.0^13,14,19,42^ and MDS 3.0^29^) and updated version, the interRAI Long-Term Care Facility (iLTCF),^4,5,12,17,20,21,26,27,29,33,37,40^ were reported on.

Instruments for use in hospitals were also featured. Eight papers focused on acute care (iAC)^4,13,21,26-28,30,33^ and three on emergency departments (iED^39^ and iED Screener^4,41^).

A number of papers focused on mental health instruments, including inpatient (n = 6; RAI-MH^7,8,13,17^ and iMH^4,24,25^) and community (n = 4; iCMH^4,13,24,25^). Among these, two also reported on use of supplements for forensic psychiatry,^4,25^ addictions,^4,25^ and intellectual and developmental disabilities.^4^ The interRAI Intellectual Disabilities (iID) was featured in three papers,^4,13,36^ while the instruments for Children and Youth Mental Health (iChYMH)^9^ and Brief Mental Health Screener (iBMHS)^33^ were each featured in one paper. The Quality of Life (iQoL) assessment was highlighted in one paper.^4^

While most focused on a single interRAI instrument^5-11,14-16,18,19,21-23,28,30,31,34-36,38-43^ (n = 27), some included multiple instruments used within or across different sectors. Six papers considered two assessments^20,24,25,29,32,37^ and six reported on three instruments.^12,17,18,26,27^ Three papers reviewed four or more instruments across a continuum of care.^4,13,33^

#### Implementation Status

There were eight papers that discussed interRAI assessments that had already been implemented^14,17,19,26,33,37,38,40,43^ and 23 described pilot or new implementations.^4,6-11,15,16,18,20-25,27,28,30-32,36,42^ Four papers did not recommend implementation of interRAI instruments or applications, namely: the RAI-HC in the Department of Veterans Affairs,^34^ the interRAI Frailty Scale in a home meal delivery service in the United States,^35^ and the iED.^39,41^ One Australian paper recommended implementation of the iHC and iLTCF after a systematic search and evaluation of comprehensive geriatric assessments but the government had yet to decide whether to implement, where another paper conducted a feasibility analysis for implementing the interRAI suite of comprehensive functional assessments in the Australian aged care system, addressing barriers and pathways for systemic adoption.^5^ One paper did not focus on implementation per se but rather highlighted the utility of multiple interRAI assessment systems.^13^ Another compared interRAI assessments to others used in a Canadian province (i.e., Québec) but did not explicitly make a recommendation.^12^

### Synthesis of Results

This section summarizes themes related to strengths and barriers related to implementation of interRAI assessments identified in the eligible papers (see Table 1).

**Table 1.** Summary of Strengths and Barriers to Implementation

Strengths	Key elements	Studies (reference #)
Assessment instruments	Comprehensive	4,7,10,13,15,17,18,20,23-26,28,30,32–34,38,40
	Standardized	4,5,7,9,13,15,16,19,21,23–26,32,37
	Clinical relevance	8,12,13,15,16,18,19,22,25,27,28,35
	Compatibility across sectors	4,5,10,11,13,24,26,28,29,33,34
	Psychometric properties	10,13,24,25,27–29,36,38,39
	Quality of data	8,10,30
	Cultural sensitivity	27,31
Assessment outputs and applications	Multiple uses of information	6–11,13,29,32,42
	Care planning	4,13,24,28,30,37,42
	Case-mix	4,7,8,11,13–15,42
	Quality of care	12,13,24,40,42,43
	Decision support	7,24,29
	Monitoring over time	15,28,42
	Policy-making	4,42,43
	System-level comparisons	4,7
Process	Enhanced collaboration/coordination	4,18,19,21,23,24,28,32,37,38,40
	Reduced assessment time/burden	18,28,38
Infrastructure	Software	4,10,19,21,26,27,30,32,37,38,40
Barriers	Key elements	Studies (reference #)
Assessment instruments	Complexity of items/coding	15,18,20,25,27
	Clinical relevance/gaps	10,21,22,25
	Psychometric properties	28,39,41
Process	Length of time to complete	7,8,15,20,22–25,28,32,33,37,40
	Not fully understanding/using applications	13,19,20,22,25,32,33,37,42,43
	Overlap with existing assessments/records	13,20,32–34,42
	Lack of collaboration	4,21,22,37
	Lack of role clarity	10,37,42
	Insufficient information on person	18,21
Infrastructure	Technological infrastructure	4,5,11,13,19,20,23–26,28–30,32–35,39,40,42,43
	Insufficient/problematic technology	19,22,25,28,30,32,39,40,43
	Lack of interoperability with existing technology	4,24,26,33,35
	Lack of education/training	11,19,24,29,32–34
	High staff workload	21,26
Staff-related	Staff resistance	28,40
	Fear of loss of clinical autonomy	4,37
	Culture change	29,30,42
	Privacy concerns	13,16

#### Strengths for Implementation

Several papers noted the comprehensive^4,7,10,13,15,17,18,20,23-26,28,30,32-34,38,40^ and standardized^4,5,7,9,13,15,16,19,21,23-26,32-37^ nature of instruments and their solid psychometric properties^10,13,24,25,27-29,36,38,39^ as strengths. Clinical relevance of items and applications,^8,12,13,15,16,18,19,22,25,27,28,35^ cultural sensitivity,^27,31^ data quality,^8,12,30^ and availability of software^4,10,19,21,26,27,30,32,37,38,40^ were also noted as strengths. Papers also noted that implementation contributed to overall reduction of assessment time or burden^18,28,38^ and enhanced collaboration and coordination^4,18,19,21,23,24,28,32,37,38,40^ within settings and across sectors. The benefit of an integrated suite and resulting compatibility across sectors was also highlighted.^4,5,10,11,13,24,26,28,29,33,34^

Many studies saw strengths in specific applications of assessment information, including decision support,^7,24,29^ care planning,^4,13,24,28,30,37,42^ case-mix,^4,7,8,11,13-15,42^ monitoring over time,^15,28,42^ system-level comparisons,^4,7^ and policy-making^4,42,43^; a few noted the ability to improve quality of care^12,13,24,40,42,43^ and access to services^9^ from the assessment data. Several explicitly noted the multiple uses of assessment information^6,7,9-11,13,29,32,38,42^ as a strength for implementation.

#### Barriers to Implementation

The most frequently cited barrier to implementation was a lack of infrastructure,^4,5,9,11,19,21-24,28-30,32-34,39,42,43^ in particular related to lack of technological infrastructure^4,5,11,13,19,20,23-26,28-30,32-35,39,40,42,43^ and education/training.^11,19,24,29,32-34^ Insufficient or problematic technology^19,22,25,28,30,32,39,40,43^ and lack of interoperability with existing electronic systems^4,24,26,33,35^ were also highlighted.

Characteristics of the assessments were also named as barriers, including perceived length/time to complete,^7,8,15,20,22-25,28,32,33,37,40^ perceived complexity of some of the items/coding,^15,18,20,25,27^ poor psychometric properties,^28,39,41^ gaps in terms of clinically important issues,^10,21,22,25^ and overlap with existing assessments or records.^13,20,32-34,42^

A lack of role clarity^10,37,42^ and collaboration^4,21,22,37^ in completing assessments, high staff workload,^21,26^ and insufficient information to complete the assessment^18,21^ were also noted barriers. Staff resistance,^28,40^ fear of loss of clinical autonomy via use of a standardized assessment,^4,37^ culture change,^29,30,42^ and privacy concerns related to data sharing^13,16^ were also noted as barriers. That clinicians or organizations did not fully understand or use all applications from the assessment was often listed as a barrier.^13,19,20,22,25,32,33,37,42,43^

## Discussion

This review identified several strengths and barriers to the process of implementing interRAI instruments, based on papers focused on systems that had previously been implemented, and on pilot studies and new implementations. That the vast majority had implemented signifies that these barriers, while challenging, are surmountable.

From these papers, several lessons can be learned to facilitate successful implementation of interRAI systems. There is a need for sufficient allocation of resources to ensure adequate training of staff,^4,6,8-14,16,18-20,22-26,28-32,35,38,40,42,43^ not only on completion of the assessment but on the use of the information it generates to support clinical decision-making and service planning and provision. For example, in Canada, which has implemented multiple interRAI assessments, the Canadian Institute for Health Information (CIHI) provides support for organizational and jurisdictional training prior to implementation^44^ and utilizing the train-the-trainer model supports ongoing training of interRAI systems.^45^ As well, many provinces also utilize e-learning and competency testing products to support their ongoing training needs. Another important allocation of resources required for successful implementation relates to information technology equipment and software.^11,16,23,27^

There is also a need to eliminate the perceived burden of assessment by realigning assessment practices—for example, by eliminating other assessments or data collection that are redundant, ensuring interRAI assessments are seamlessly incorporated into the care process and the electronic clinical records, and that the information can be shared within and across settings supporting individuals. To this end, there is a need for investment in information and technology solutions that support not only completion of the assessment, but also use of the outputs (e.g., care planning protocols, risk algorithms, and outcome measures) in real time. Further to this, software needs to synthesize patient complexity and be presented in a usable manner to the care providers in order to support care planning and emphasize patient’s needs.^46^ User-friendly outputs that can support care planning and decision-making from the local to national level are also important. Investment in technology infrastructure was recommended in several papers.^4,7,12,13,16,19-24,26-33,35,37,38,40,42^

Technology infrastructure is a vital component of the interRAI system as it facilitates the seamless flow of data across different systems, promoting continuity of care and improving the quality and effectiveness of health and social services. As an example, BelRAI is a centralized web-based platform in Belgium that uses the interRAI assessment system to facilitate the transfer of assessment information between different care settings (e.g., home care, nursing home, palliative care, and mental healthcare), promoting seamless transitions and continuity of care. Moreover, the BelRAI infrastructure provides a secure on-line platform for sharing assessment data, ensuring privacy and confidentiality.^4,26,47^

A few studies recommended evaluation of interRAI systems^7^ and an organizational approach^12,34,38^ to implementation, whereby it is recommended that organizational goals be considered in the implementation.

The most successful implementations are ones that can leverage interRAI assessments to support decision-making at the levels of the individual, organization, and system. To this end, government and organizational champions, leadership, and support are important,^4,5,9,12,17,19,25,26,30,34,35,37,38^ as is a clear communication strategy.^4,9-11,16,26,30^ Furthermore, mandated implementations are often identified as critical to implementation.^9,17,23,29^ For example, in Australia, a paper recommended interRAI instruments for aged care but recognized that a government mandate would be necessary for successful implementation of interRAI systems.^5^

Strategic engagement and interprofessional involvement (e.g., government officials, policy-makers, stakeholders, clinicians, and managers) were also noted as powerful facilitators both in the initial implementation and with ongoing use of interRAI systems.^4,16,20,32,37,42^

The recommendations outlined in this article provide a summary of the lessons learned from several countries using a variety of interRAI assessments from pilot studies to national implementations. The lessons learned from the implementation of interRAI systems provide a foundation to support others who wish to pursue implementation outlining the key factors to take into consideration when developing an implementation strategy.

## Conclusion

The papers analyzed in this rapid review identified several benefits to interRAI system implementations. Despite the existence of challenging barriers, there have been many successful implementations which suggest that the barriers can be overcome and the benefits of implementation of interRAI system are seen as worthy of the effort to overcome these barriers.

This rapid review provides an overview of papers that shed light on the strengths and barriers found with the implementation of interRAI assessments across care settings and across the age continuum. With this information, we can identify ways to help enhance the strengths and mitigate the barriers associated with implementation to help ease the implementation process for sites, organizations, governments, and policymakers wishing to engage or are engaged in interRAI suit implementation.

### Limitations

A potential limitation to this rapid review could be conducting a systematic literature search in PubMed only may limit the peer-reviewed articles towards the inclusion of more medically based literature. However, this may be warranted given interRAI systems are implemented across healthcare settings both in hospital and in the community. Furthermore, the incorporation of international reports on pilot studies and implementation of interRAI assessment instruments expanded the scope of content included in this rapid review. Another potential limitation could be that a critical appraisal of the papers included in the rapid review was not conducted. However, given the overwhelming agreement in terms of themes, we are confident that these are reflective of the implementations studied.

More information about the data extraction is available by contacting kmathias@uwaterloo.ca.
